# Prevalence, Correlates, and Time Trends of Multiple Chronic Conditions Among Israeli Adults: Estimates From the Israeli National Health Interview Survey, 2014–2015

**DOI:** 10.5888/pcd14.170038

**Published:** 2017-08-10

**Authors:** Samah Hayek, Anneke Ifrah, Teena Enav, Tamy Shohat

**Affiliations:** 1 Israel Center for Disease Control, Israel Ministry of Health, Jerusalem, Israel; 2 Department of Epidemiology and Preventive Medicine, Tel Aviv University, Tel Aviv, Israel

## Abstract

**Introduction:**

Chronic diseases constitute a major public health challenge. The prevalence of multiple chronic conditions (MCC) has increased. The objective of our study was to describe the prevalence, correlates, and time trends of MCC in the Israeli population and among the nation’s 2 main population groups (Jewish and Arab).

**Methods:**

To describe the prevalence of correlates of MCC, we used data from the 2014–2015 Israeli National Health Interview Survey-III (INHIS-III). MCC was defined as having 2 or more of the following 10 self-reported physician-diagnosed chronic conditions: asthma, arthritis, cancer, diabetes, dyslipidemia, heart attack, hypertension, migraine, osteoporosis, or thyroid disease. For trend analysis, we used data from INHIS-I (2003–2004) and INHIS-II (2007–2010). Logistic regression was used for multivariate analysis. Estimates were weighted to the 2014 Israeli population. *P* for trend was calculated by using the Cochran–Armitage test for proportions.

**Results:**

In 2014–2015, the prevalence of MCC was 27.3% (95% confidence interval, 25.7%–28.8%). In multivariate analysis, MCC was associated with older age, female sex, a monthly household income of USD$3,000 or less, current and past smoking, and overweight or obesity. After adjusting for age, sex, income, smoking status, and body mass index, differences in MCC between Jewish and Arab populations disappeared. Dyslipidemia and hypertension were the most prevalent dyad among both men and women. Dyslipidemia, hypertension, and diabetes were the most prevalent triad among both men and women. The age-adjusted prevalence of MCC increased by 6.7% between 2003–2004 and 2014–2015.

**Conclusion:**

With the increase in the prevalence of MCC, a comprehensive approach is needed to reduce the burden of chronic conditions. Of special concern are the groups most prone to MCC.

## Introduction

Chronic or noncommunicable diseases are a major public health challenge and result in 38 million deaths worldwide each year (1). The proportion of mortality attributed to chronic conditions worldwide is expected to increase from 59% in 2002 to 69% in 2030 (2). In the United States, the prevalence of chronic conditions is rising, and by 2020 an estimated 157 million Americans will have at least 1 chronic condition (3,4). In recent years, the number of people living with multiple chronic conditions (MCC) has increased (4). MCC is defined by the US Department of Health and Human Services as the presence of 2 or more chronic conditions (5). In the United States, the prevalence of MCC increased from 21.8% in 2001 to 26% in 2010 (3,6), and an estimated 71% of all health care spending is allocated to the care of people with MCC (7). In recent years, the number of studies on the management and treatment of people with MCC has also increased (8); people with MCC constitute a growing health care and financial burden on the health system (9). MCC is more often present among the older population, among those with lower levels of education, among people living alone or in a home for the elderly (10), and among those living in deprived areas (11).

The primary objectives of our study were to describe the prevalence and correlates of MCC among the Israeli population overall and to compare MCC in the nation’s 2 main population groups (Jewish and Arab). A secondary objective was to examine time trends in MCC.

## Methods

We used data from the Israeli National Health Interview Survey (INHIS), a cross-sectional population-based telephone survey conducted periodically by the Israel Center for Disease Control. The main source of data for our analysis was the most recent INHIS survey, INHIS-III, conducted in 2014–2015 on a random representative sample of 4,325 Israeli adults. For the trend analysis, we used data from the previous 2 surveys, INHIS-I (2003−2004) and INHIS-II (2007–2010), which used the same methodology in data collection and sampling as INHIS-III. Data collection and procedures of INHIS-I are detailed elsewhere (12). The survey questionnaire is based on the European Health Interview Survey framework initiated by the World Health Organization (WHO) Regional Office for Europe in 2000 (13).

For the INHIS-III, a random sample of telephone numbers of 19,692 Jewish households and 10,799 Arab households was extracted from a computerized list of all household landlines in Israel. Inclusion criteria included households with residents aged 21 years or older, who were able to communicate in Hebrew in the Jewish sample or Arabic in the Arab sample. In 4,325 households an eligible resident was contacted and interviewed. Oral informed consent was obtained from each participant.

### Definitions

Chronic conditions were assessed by asking the participant whether he or she had ever received a diagnosis from a physician for any of the following 10 conditions: asthma, arthritis, cancer, diabetes, dyslipidemia, heart attack, hypertension, migraine, osteoporosis, or thyroid disease. Persons who responded yes to having a physician-diagnosed chronic condition were considered to have a chronic condition. MCC was defined as having 2 or more self-reported physician-diagnosed chronic conditions (3,4,6).

The questionnaire included questions on sociodemographic characteristics (age, sex, population group, marital status, monthly household income [in US dollars], and number of years of schooling) and smoking status (never smoked, past smoker, or current smoker). Body mass index (BMI) was calculated by dividing reported weight by the square of reported height (kg/m^2^) and categorized, according to WHO guidelines, as underweight, normal weight, overweight, and obese (14). Physical activity was calculated according to WHO guidelines for physical activity, which recommend at least 150 minutes of moderate-intensity aerobic physical activity per week (15).

### Statistical analysis

For the descriptive analysis, we calculated prevalence and 95% confidence intervals (CIs) for chronic conditions and MCC. We calculated the weighted prevalence of chronic conditions; weights were calculated from the general population for each year of the survey: INHIS-I (2004), INHIS-II (2010), and INHIS-III (2014).

We conducted bivariate analysis to explore associations between MCC and sociodemographic characteristics, household income, smoking status, and BMI; the χ^2^ test was used for categorical variables. We conducted multivariate analysis using a weighted logistic regression model and calculated adjusted prevalence rate ratios (PRRs) and 95% CIs. We examined time trends in MCC by comparing the age-adjusted prevalence of MCC in INHIS-III with the age-adjusted prevalence of MCC in INHIS-I and INHIS-II (16,17). The population of Israel in 2010 was used as the standard population to estimate the age adjusted rates of MCC. *P* for trend was calculated by using the Cochran–Armitage trend test for proportions. We did not include thyroid disease in the trend analysis because it was not included in INHIS-I. Percentage change was calculated as the difference between INHIS-III and INHIS-I rates, divided by the INHIS-I rate and multiplied by 100. The number of chronic conditions was grouped into 4 categories: none, 1 chronic condition, 2 or 3 chronic conditions, and 4 or more chronic conditions.

Finally, we conducted a sensitivity analysis using the following 5 clusters to explore prevalence and trends: 1) asthma or migraine; 2) hypertension, dyslipidemia, heart attack, or diabetes; 3) arthritis or osteoporosis; 4) cancer; and 5) thyroid disease. We conducted all statistical analyses using SAS version 9.1 (SAS Institute, Inc). A *P* value of <.05 was considered significant.

## Results

In INHIS-III, 69.3% of respondents were Jewish and 30.7% were Arab. The average age was 47.2 years (standard deviation, 16.3 y), with a median age of 53; most respondents were married or living with a partner (80.7%); 50.4% were men; 55.5% had completed more than 12 years of schooling; 37.6% reported a monthly household income of $2,000 or less; 19.6% were current smokers, 22.1% were past smokers, and 58.3% had never smoked; 21% had a BMI of 30.0 or more; and 33.8% reported at least 150 minutes per week of physical activity.

### Prevalence and correlates of chronic conditions and MCC

In INHIS-III, 54.3% (95% CI, 52.3%–56.3%) of respondents reported at least 1 chronic condition. The prevalence of MCC was 27.3% (95% CI, 25.7%–28.8%) (Table 1). In the bivariate analysis, the prevalence of MCC was significantly associated with older age, female sex, being Jewish, having a monthly household income of $2,000 or less or a monthly household income of $2,001 to $3,000, having 12 years of schooling or fewer, current or past smoking, and overweight or obesity (Table 1). After adjusting for age, prevalence rates of MCC were higher among the Arab population than in the Jewish population in INHIS-I, INHIS-II, and INHIS-III (Figure).

**Table 1:** Prevalence of Multiple Chronic Conditions (≥2 Chronic Conditions) Among Israelis Aged ≥21 Years, by Selected Demographic Characteristics, Israeli National Health Interview Survey, 2014–2015

Characteristic	No. (Weighted %) [95% CI]	*P *Value^a^
**Total**	1,579 (27.3) [25.7–28.8]	—
**Age, y**		
21–34	40 (8.3) [5.6–11.0]	<.001
35–49	234 (16.2) [14.1–18.2]	
50–64	544 (40.6) [37.7–43.5]	
≥65	761 (64.1) [61.3–66.9]	
**Sex**		
Male	726 (23.2) [21.2–41.2]	<.001
Female	853 (31.2) [28.9–33.5]	
**Population group**		
Jewish	1,127 (27.9) [26.1–29.7]	.02
Arab	452 (24.4) [22.7–26.7]	
**Monthly household income, US$**		
≤2,000	243 (41.3) [35.4–47.3]	<.001
2,001–3,000	744 (27.9) [25.7–30.3]	
3,001–4,000	159 (21.0) [17.6–24.4]	
>4,000	191 (23.7) [20.1–27.4]	
**Years of schooling**		
>12	751 (23.5) [21.6–25.4]	<.001
≤12	801 (33.3) [30.8–35.8]	
**Marital status**		
Married or living with a partner	1,263 (27.4) [25.7–29.1]	.32
Unmarried	316 (27.2) [23.7–30.6]	
**Smoking status**		
Never	829 (23.9) [22.0–25.7]	.001
Current	276 (26.0) [22.5–29.5]	
Past	454 (39.4) [35.8–42.9]	
**BMI^b^**		
Underweight (18.5)	10 (16.5) [4.0–28.9]	<.001
Normal weight (18.5 to <25.0)	377 (18.1) [15.9–20.2]	
Overweight (25.0 to ≤29.9)	627 (30.9) [28.4–33.4]	
Obese (≥30.0)	422 (42.7) [38.6–46.9]	
**Physical activity^c^**		
<150 min per week	1,050 (27.3) [25.4–29.1]	.72
≥150 min per week	529 (27.4) [24.9–29.9]	

Abbreviations: —, not applicable; BMI, body mass index; CI, confidence interval.

a Determined by weighted bivariate analysis.

b Calculated by dividing reported weight by the square of reported height (kg/m^2^) (14).

c Physical activity guidelines of World Health Organization (15).

**Figure:**
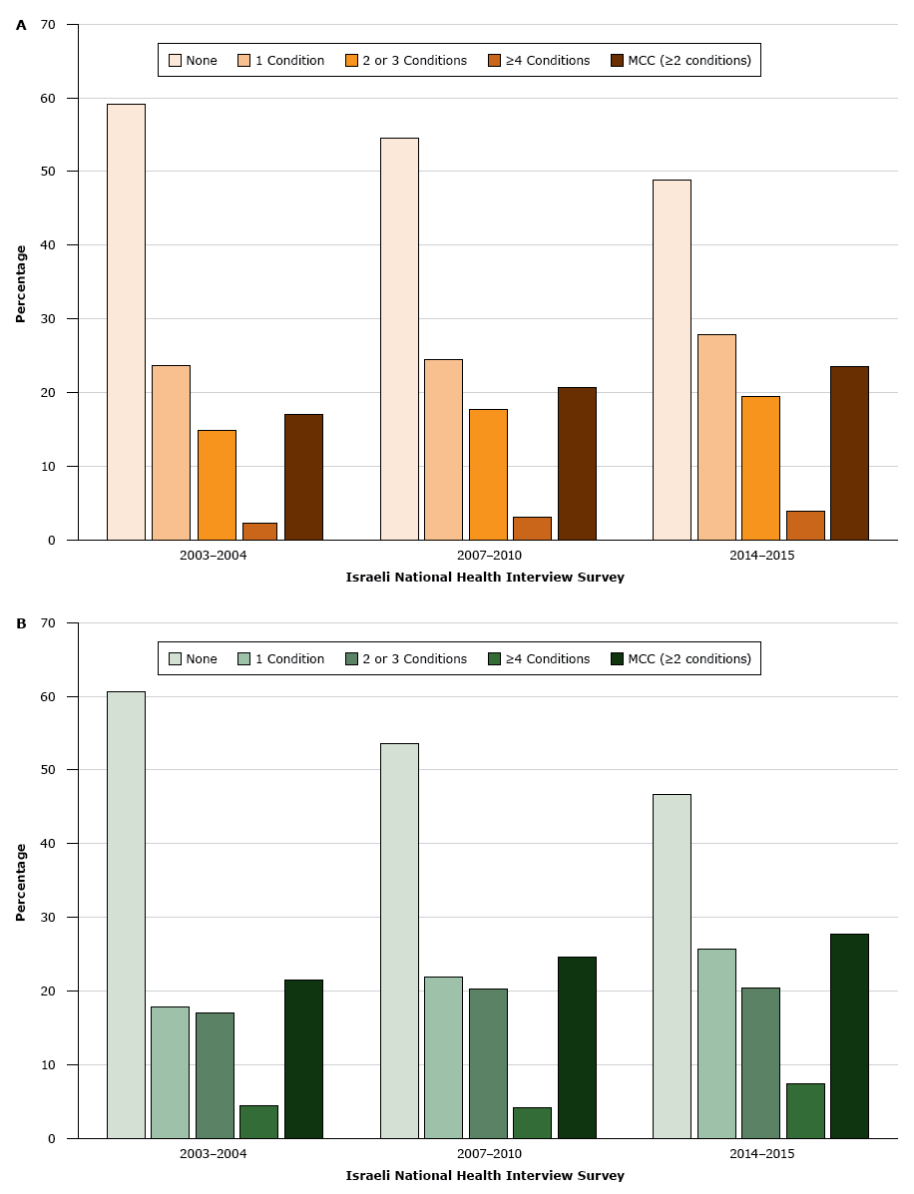
Age-adjusted prevalence of chronic conditions in the A) Jewish population and B) Arab population in Israel, by number of chronic conditions, Israeli National Health Interview Survey, 2003–2004, 2007–2010, and 2014–2015. *P* for trend was <.05 for all chronic conditions and for multiple chronic conditions (MCC). MCC was defined as 2 or more chronic conditions. **Table:** 

Survey Year	None, %	1 Condition, %	2 or 3 Conditions, %	≥4 Conditions, %	MCC (≥2 Conditions), %
Jewish population					
2003–2004	59.1	23.7	14.8	2.2	17.0
2007–2010	54.5	24.4	17.7	3.0	20.6
2014–2015	48.8	27.8	19.5	3.9	23.5
Arab population					
2003–2004	60.6	17.8	17.0	4.5	21.5
2007–2010	53.4	21.9	20.3	4.2	24.6
2014–2015	46.5	25.7	20.4	7.4	27.8

In the multivariate logistic regression, MCC was associated with older age, female sex, a monthly household income of $3,000 or less, current or past smoking, and overweight or obesity. The risk for MCC was 17 times higher among respondents aged 65 or older than among respondents aged 21 to 34. It was higher among women (adjusted PRR, 1.6) than among men, among respondents with a monthly household income of $2,000 or less (adjusted PRR, 1.7) or $2,001 to $3,000 (adjusted PRR, 1.3) than among respondents with a monthly household income of more than $4,000, among current smokers (adjusted PRR, 1.4) and past smokers (adjusted PRR, 1.3) than among nonsmokers, and among overweight (adjusted PRR, 1.9) and obese (adjusted PRR, 3.0) respondents than among normal-weight respondents (Table 2).

**Table 2:** Factors Associated With Multiple Chronic Conditions (≥2 Chronic Conditions) Among Israelis Aged ≥21 Years, Israeli National Health Interview Survey, 2014–2015^a^

Characteristic	Adjusted Prevalence Rate Ratios (95% CI)	*P *Value
**Age, y**		
21–34	1 [Reference]	
35–49	2.1 (1.6–2.8)	<.001
50–64	6.5 (4.9–8.7)	<.001
≥65	17.0 (12.6–23.1)	<.001
**Sex**		
Male	1 [Reference]	
Female	1.6 (1.3–2.0)	<.001
**Population group**		
Jewish	1 [Reference]	
Arab	1.0 (0.8–1.3)	.72
**Monthly household income, US$**		
≤2,000	1.7 (1.2–2.5)	.005
2,001–3,000	1.3 (1.0–1.7)	.04
3,001–4,000	1.0 (0.7–1.3)	.83
>4,000	1 [Reference]	
**Years of schooling**		
<12	1 [Reference]	
≥12	1.0 (0.8–1.2)	.59
**Marital status**		
Married or living with a partner	1 [Reference]	
Unmarried	1.1 (0.9–1.4)	.22
**Smoking status**		
Never	1 [Reference]	
Current	1.4 (1.1–1.8)	<.001
Past	1.3 (1.0–1.7)	.01
**BMI^b^**		
Normal weight (18.5 to <25.0)	1 [Reference]	
Underweight (<18.5)	1.0 (0.5–1.9)	.91
Overweight (25.0 to ≤29.9)	1.9 (1.5–2.2)	<.001
Obese (≥30.0)	3.0 (2.4–3.9)	<.001
**Physical activity^c^**		
<150 min per week	0.9 (0.7–1.1)	.21
≥150 min per week	1 [Reference]	

Abbreviations: BMI, body mass index; CI, confidence interval.

a Determined by weighted multivariate logistic regression.

b Calculated by dividing reported weight by the square of reported height (kg/m^2^) (14).

c Physical activity guidelines of World Health Organization (15).

### Most prevalent chronic conditions and combinations (dyads and triads)

In 2014, the most prevalent chronic conditions were dyslipidemia (29.6%; 95% CI, 28.3%–31.4%), hypertension (20.6%; 95% CI, 19.3%–21.9%), thyroid disease (8.9%; 95% CI, 7.9%–9.9%), migraine (8.8%; 95% CI, 7.6%–9.9%), diabetes (9.3%; 95% CI, 8.4%–10.1%), asthma (7.2%; 95% CI, 6.5%–8.5%), and arthritis (5.5%; 95% CI, 4.8%–6.2%). Our sensitivity analysis indicated that the most prevalent disease cluster was hypertension, dyslipidemia, heart attack, or diabetes (40.5%; 95% CI, 38.7%–42.3%).

The most prevalent dyad was dyslipidemia and hypertension (24.0%; 95% CI, 20.7%–27.3%) (Table 3), and the most prevalent triad was dyslipidemia, hypertension, and diabetes (17.9%; 95% CI, 13.9%–21.9%). In the analysis of dyads by sex, the most prevalent dyad was dyslipidemia and hypertension for both men (31.8%; 95% CI, 26.5%–37.2%) and women (17.5%; 95% CI, 13.5%–21.5%). The most prevalent triad was dyslipidemia, hypertension, and diabetes for both men (29.5%; 95% CI, 21.7%–37.2%) and women (10.9%; 95% CI, 6.7%–15.0%). The most prevalent dyad of disease clusters was asthma or migraine/hypertension, dyslipidemia, heart attack, or diabetes (31.5%; 95% CI, 27.9%–35.2%) (Table 4). The most prevalent triad of disease clusters was asthma or migraine/hypertension, dyslipidemia, heart attack, or diabetes/arthritis or osteoporosis (28.4%; 95% CI, 21.2%–35.7%)

**Table 3:** Prevalence of the 5 Most Prevalent Dyads and Triads of Multiple Chronic Conditions (≥2 Chronic Conditions) Among Israelis Aged ≥21 Years, by Sex, Israeli National Health Interview Survey, 2014–2015

Dyad or Triad, by Sex	Weighted % (95% Confidence Interval)
**Dyads**	
**Overall**	
Dyslipidemia and hypertension	24.0 (20.7–27.3)
Dyslipidemia and thyroid disease	6.6 (4.7–8.6)
Dyslipidemia and migraine	6.1 (4.2–7.9)
Dyslipidemia and diabetes	5.9 (4.1–7.8)
Dyslipidemia and asthma	5.8 (4.0–7.7)
**Men**	
Dyslipidemia and hypertension	31.8 (26.5–37.2)
Dyslipidemia and asthma	10.0 (6.5–13.5)
Dyslipidemia and diabetes	7.7 (4.6–10.8)
Dyslipidemia and cancer	4.6 (2.2–7.1)
Diabetes and hypertension	4.0 (1.7–6.3)
**Women**	
Dyslipidemia and hypertension	17.5 (13.5–21.5)
Dyslipidemia and thyroid disease	9.2 (6.1–12.2)
Dyslipidemia and migraine	8.1 (5.3–11.1)
Dyslipidemia and arthritis	4.5 (2.3–6.7)
Dyslipidemia and diabetes	4.4 (2.3–6.6)
**Triads**	
**Overall**	
Dyslipidemia, hypertension, and diabetes	17.9 (13.9–21.9)
Dyslipidemia, hypertension, and thyroid disease	7.0 (4.4–9.7)
Dyslipidemia, hypertension, and heart attack	5.3 (2.9–7.7)
Dyslipidemia, hypertension, and osteoporosis	4.7 (2.5–7.0)
Dyslipidemia, hypertension, and cancer	4.5 (2.3–6.7)
**Men**	
Dyslipidemia, hypertension, and diabetes	29.5 (21.7–37.2)
Dyslipidemia, hypertension, and heart attack	12.7 (7.0–18.4)
Dyslipidemia, hypertension, and cancer	8.5 (3.7–13.3)
Dyslipidemia, hypertension, and asthma	3.1 (0.1–6.0)
Dyslipidemia, diabetes, and asthma	3.0 (0.1–3.9)
**Women**	
Dyslipidemia, hypertension, and diabetes	10.9 (6.7–15.0)
Dyslipidemia, hypertension, and thyroid disease	10.3 (6.3–14.4)
Dyslipidemia, hypertension, and osteoporosis	7.0 (3.6–10.4)
Dyslipidemia, hypertension, and migraine	5.0 (2.1–7.9)
Dyslipidemia, hypertension, and arthritis	3.1 (0.7–5.4)

**Table 4:** Prevalence of the 5 Most Prevalent Dyads and Triads of Clusters of Chronic Conditions (≥2 Chronic Conditions) Among Israelis Aged ≥21 Years, by Sex, Israeli National Health Interview Survey, 2014–2015

Clusters, by Sex	Weighted % (95% Confidence Interval)
**Dyads**	
**Overall**	
Asthma or migraine/hypertension, dyslipidemia, heart attack, or diabetes	31.5 (27.9–35.2)
Hypertension, dyslipidemia, heart attack, or diabetes/arthritis or osteoporosis	25.5 (22.1–28.9)
Hypertension, dyslipidemia, heart attack, or diabetes/thyroid disease	17.5 (14.5–20.5)
Hypertension, dyslipidemia, heart attack, or diabetes/cancer	12.5 (9.6–14.8)
Asthma or migraine/thyroid disease	4.5 (2.8–6.2)
**Men**	
Asthma or migraine/hypertension, dyslipidemia, heart attack, or diabetes	39.7 (33.3–46.1)
Hypertension, dyslipidemia, heart attack, or diabetes/cancer	22.6 (17.2–28.1)
Hypertension, dyslipidemia, heart attack, or diabetes/arthritis or osteoporosis	19.3 (14.2–24.5)
Hypertension, dyslipidemia, heart attack, or diabetes/thyroid disease	12.2 (7.9–16.5)
Asthma or migraine/arthritis or osteoporosis	1.9 (1.1–3.6)
**Women**	
Hypertension, dyslipidemia, heart attack, or diabetes/arthritis or osteoporosis	29.3 (24.6–33.5)
Asthma or migraine/hypertension, dyslipidemia, heart attack, or diabetes	26.9 (22.5–31.2)
Hypertension, dyslipidemia, heart attack, or diabetes/thyroid disease	20.6 (16.6–24.5)
Asthma or migraine/thyroid disease	6.8 (4.3–9.3)
Asthma or migraine/arthritis or osteoporosis	6.4 (4.3–9.3)
**Triads**	
**Overall**	
Asthma or migraine/hypertension, dyslipidemia, heart attack, or diabetes/arthritis or osteoporosis	28.4 (21.2–35.7)
Hypertension, dyslipidemia, heart attack, or diabetes/arthritis or osteoporosis/thyroid disease	22.6 (15.9–29.4)
Asthma or migraine/hypertension, dyslipidemia, heart attack, or diabetes/thyroid disease	15.8 (9.9–21.6)
Asthma or migraine/hypertension, dyslipidemia, heart attack, or diabetes/cancer	12.2 (6.9–17.4)
Hypertension, dyslipidemia, heart attack, or diabetes/arthritis or osteoporosis/cancer	11.7 (6.6–16.9)
**Men**	
Asthma or migraine/hypertension, dyslipidemia, heart attack, or diabetes/cancer	22.4 (6.1–37.9)
Asthma or migraine/hypertension, dyslipidemia, heart attack, or diabetes/arthritis or osteoporosis	21.3 (6.0–36.5)
Asthma or migraine/hypertension, dyslipidemia, heart attack, or diabetes/thyroid disease	18.9 (4.3–33.4)
Hypertension, dyslipidemia, heart attack, or diabetes/arthritis or osteoporosis/cancer	13.7 (0.8–26.7)
Hypertension, dyslipidemia, heart attack, or diabetes/cancer/thyroid disease	11.6 (0.0–21.6)
**Women**	
Asthma or migraine/hypertension, dyslipidemia, heart attack, or diabetes/arthritis or osteoporosis	30.0 (21.9–38.2)
Hypertension, dyslipidemia, heart attack, or diabetes/arthritis or osteoporosis/thyroid disease	25.5 (17.7–33.3)
Asthma or migraine/hypertension, dyslipidemia, heart attack, or diabetes/thyroid disease	15.1 (8.7–21.4)
Hypertension, dyslipidemia, heart attack, or diabetes/arthritis or osteoporosis/cancer	11.8 (6.1–17.6)
Asthma or migraine/hypertension, dyslipidemia, heart attack, or diabetes/cancer	9.3 (4.1–14.5)

### Trend analysis for MCC and chronic conditions

The prevalence of MCC increased with each administration of the INHIS among both Jewish and Arab populations, and the prevalence of having no chronic conditions correspondingly decreased (Figure). The age-adjusted prevalence of MCC increased by 6.7% between 2003–2004 and 2014–2015. Among the Arab population, the age-adjusted prevalence of MCC increased from 21.5% in 2003–2004 to 24.6% in 2007–2010 and to 27.8% in 2014–2015 (*P* < .001). Among the Jewish population, the age-adjusted prevalence of MCC increased from 17.0% in 2003–2004 to 20.6% in 2007–2010 and to 23.5% in 2014–2015 (*P* < .001). An increase was observed among all age groups (Appendix Table 1). The prevalence of each chronic condition changed significantly over time, except for arthritis and osteoporosis (Appendix Table 2). The chronic condition with the greatest increase in prevalence was cancer (a 76.0% increase), followed by dyslipidemia (a 75.1% increase). The disease cluster with the greatest increase in prevalence (a 44.6% increase) was dyslipidemia, hypertension, heart attack, and diabetes.

## Discussion

Our study explored prevalence, trends, and factors associated with MCC in Israel. The weighted prevalence of MCC in 2014–2015 was 27.3%. Although the prevalence of having 1 chronic condition in our study (24.4%) was almost similar to the prevalence in a US study in 2012 (22.3%) (18), the prevalence of MCC was lower in our study (27.3%) than in the US study (33.8%). The prevalence of MCC was lower in our study also than the prevalence (37.2%) in a study conducted in Yorkshire, England, between 2010 and 2012, among a population aged 24 or older (11). A study in Hong Kong in 2012 found a prevalence of MCC of 13.4%; this low rate may have been partly due to various levels of accessibility to health services of various social strata (19). In addition, each of these studies used a different list of chronic conditions to estimate MCC, and each used a different method of data collection, which might have affected the results. For example, higher estimates of MCC in some countries may have resulted from including chronic conditions such as fatigue and insomnia, which we did not include.

We found that MCC was associated with older age, female sex, a monthly household income of $3,000 or less, being a current or past smoker, and being overweight or obese. The association with older age is consistent with findings of the US National Health Interview Survey (3). In our study, a significantly greater percentage of women than men reported MCC. This finding is in accordance with findings in several other countries (3,18,20,21).The association of lower household income with MCC found in our study is also consistent with findings from other studies, such as a study in the mid-south region of the United States (22).

The age-adjusted prevalence of MCC was significantly higher among the Arab population than among the Jewish population. This finding is consistent with known population-group differences in health status and the prevalence of risk factors among the Israeli population. The prevalence of obesity and diabetes is higher among the Arab population than among the Jewish population (23,24); physical inactivity (ie, not being physically active for 20 minutes at least 3 times per week) is prevalent (77.5% in 2010) among the Arab population, and smoking rates among Arab men are high (47.2% in 2010) (16,17). In the multivariate analysis, after we controlled for various explanatory variables, the significant difference between the Jewish and Arab population in the prevalence of MCC disappeared, indicating that the observed difference may be explained by social and behavioral factors rather than biological or ethnic predisposition to disease. Population group-differences in risk factors for chronic diseases were reported in other studies; for example, the Racial and Ethnic Approaches to Community Health (REACH) 2010 risk factor survey conducted in the United States among 4 racial/ethnic minority populations (black, Hispanic, Asian/Pacific Islander, and American Indian) found that these populations had greater risks for disease compared with the general population living in the same area. The excess risks were attributed to differences in the distribution of risk factors, chronic conditions, and use of preventive services (25). As in the REACH study, our study did not find an association between ethnicity and MCC, after controlling for social and behavioral risk factors.

Our trend analysis indicated a significant increase in age-adjusted prevalence rates and in age-specific rates of MCC from 2003–2004 to 2014–2015. These trends were evident in both the Jewish and Arab populations. Because chronic conditions result from lifetime exposures and other risk factors, it follows that longer survival would increase the number of people living with chronic conditions (26). On the other hand, the increases could be attributed, at least partially, to changes in diagnostic criteria during the study period; for example, the diagnostic criteria for diabetes changed (27). In addition, our data on increases in rates of MCC over time are consistent with US data, which show a significant increase in MCC between 2001 and 2010 (3).

The increase in MCC was evident among all age groups, consistent with current research. An increasing number of young and middle-aged adults are reporting more than 1 chronic condition (28). One study found that the younger population is more likely to have clusters of associated diseases than to have isolated diseases, which may explain the high prevalence of MCC among young adults (10).

The increase in MCC may be attributed in part to increasing rates of risk factors associated with chronic conditions, such as obesity, a known risk factor for diabetes, coronary heart attack, elevated blood pressure, and certain types of cancer (29,30). The prevalence of obesity increased from 15.9% to 21.1% (an increase of 33%) among the Israeli adult population from 2003 to 2014 (16,17). In parallel, the prevalence of self-reported physician-diagnosed diabetes increased by 49.3% during the same time (16,17).

The increase in MCC may also be attributed to an increase in awareness and use of health services for the early detection of chronic conditions. For example, from 2003 to 2015, mammography screening in Israel increased by 23%, blood pressure screening by 29%, and cholesterol testing by 94% (16,17). Screening for early detection of cancer also contributes to increased incidence of cancer, which may be reflected in the rise in incidence of breast cancer among Israeli Arab women (31).

The disease combinations found in our study are different from the combinations found in other studies. The 2 most common dyads among men in our study were dyslipidemia and hypertension and dyslipidemia and asthma, whereas in the United States, the 2 most common dyads were hypertension and arthritis and hypertension and diabetes (3). We found similar differences for women. On the other hand, our study found that hypertension and dyslipidemia were the most prevalent chronic conditions that occur together with other chronic conditions, and this outcome is consistent with the findings of a study showing that hypertension and hyperlipidemia were the most prevalent chronic conditions in MCC (32). A German study among older adults found that the most common triads of the most prevalent chronic conditions were hypertension, lipid metabolism disorders, and chronic low back pain and diabetes mellitus, osteoarthritis, and chronic ischemic heart attack (33), whereas in our study, among the population aged 65 or older, we found hypertension dyslipidemia, and diabetes and hypertension, osteoporosis, and thyroid disease to be the most common triads. The differences in combinations may be attributed in part to differences in the chronic conditions that were investigated and to differences in type of data source. For example, survey data (generally self-reported) are likely to yield different combinations of chronic conditions than are clinical data.

Also worth noting, each chronic disease has a different impact on health-related quality of life and daily functioning. For example, osteoarthritis of the knee has a particularly great impact on the health-related quality of life of Chinese patients (34).

To compare the prevalence of MCC across studies, a standardized list of chronic conditions and common criteria for data sources needs to be created. Although our study adds to the growing evidence that MCC is becoming a greater burden on the health care system and that its prevalence is increasing among the young adult population, this outcome needs to be further investigated and validated by using data from health maintenance organizations in addition to self-reported survey data.

As far as we know, our study is the first study to explore the prevalence and correlates of MCC in 3 administrations of a large population-based survey in Israel. A major limitation of our study is the cross-sectional design, which precludes assumptions of causality. However, because our study had a large sample, it yielded stable estimates. Additionally, we could not assess the severity and duration of the chronic conditions or changes in conditions. On the other hand, because our study consisted of data on chronic conditions diagnosed by a physician, problems of recall bias were less likely to occur.

With the steady increase in the population aged 65 or older, the prevalence of MCC will continue to increase. A comprehensive approach is needed to reduce the burden of chronic conditions, including intervention programs targeting populations at risk. Because the increase in MCC was observed across all age groups, preventive strategies need to be tailored for the younger population as well as for the older population. In addition, because our study indicated that the most prevalent chronic conditions in Israel are hypertension and dyslipidemia, a principal focus of preventive intervention in Israel needs to be directed toward healthy lifestyle promotion. To enable the comparison of data across studies of MCC, the list of chronic conditions investigated and the definition of MCC need to be standardized.

## Appendices

**Appendix:** Table 1. Trends in Prevalence of Multiple Chronic Conditions (≥2 Chronic Conditions) Among Israelis Aged ≥21 Years, by Age Group, Israeli National Health Interview Survey (INHIS), 2003–2014

Age Group, y	INHIS-I (2003–2004), No. (Weighted %) [95% Confidence Interval]	INHIS-III (2014–2015), No. (Weighted %) [95% Confidence Interval]
21–34	53 (4.1) [3.0–5.3]	34 (7.1) [4.6–9.6]
35–49	240 (13.4) [11.7–14.9]	203 (14.0) [12.1–15.9]
50–64	698 (34.9) [32.7–37.2]	499 (36.6) [33.8–39.4]
≥65	837 (47.7) [45.2–49.8]	726 (61.0) [58.1–63.8]

**Appendix:** Table 2. Trends in the Prevalence of Chronic Conditions (≥2 Chronic Conditions) Among Israelis Aged ≥21 Years, Israeli National Health Interview Survey (INHIS)^a^

Condition or Cluster	INHIS-I (2003–2004), No. (%) (N = 9,509)	INHIS-II (2007–2010), No. (%) (N = 10,331)	INHIS-III (2014–2015), No. (%) (N = 4,351)	% Change From 2003–2004 to 2014–2015	*P* for Trend^b^
**Condition**					
Asthma	539 (5.7)	572 (5.8)	312 (7.2)	26.3	.008
Hypertension	1,636 (15.3)	2,472 (19.8)	1,196 (20.6)	34.6	<.001
Dyslipidemia	1,724 (16.9)	3,033 (24.7)	1,630 (29.6)	75.1	<.001
Heart attack	361 (3.5)	420 (2.9)	218 (2.9)	−17.1	.01
Arthritis	578 (5.2)	495 (3.7)	347 (5.5)	5.7	.08
Osteoporosis	545 (5.1)	639 (5.4)	293 (5.0)	−1.9	.17
Migraine	674 (6.8)	762 (7.0)	361 (8.8)	29.4	<.001
Diabetes	683 (6.1)	1,089 (8.3)	578 (8.4)	37.3	<.001
Cancer	256 (2.5)	448 (3.5)	257 (4.4)	76.0	<.001
**Clusters**					
Asthma or migraine	1,147 (11.9)	1,259 (12.2)	633 (15.2)	27.7	<.001
Hypertension, dyslipidemia, heart attack, or diabetes	2,933 (28.0)	4,353 (35.3)	2,249 (40.5)	44.6	.001
Arthritis or osteoporosis	993 (9.1)	1,025 (8.2)	565 (9.2)	1.1	.63
Cancer	256 (2.5)	448 (3.5)	257 (4.4)	76.0	<.001

^a^ Percentages were weighted for age, sex, and population group.

^b^ For weighted percentage.
